# Continuous administration of heparin during free flap surgery for head and neck tumors reduces the risk of pulmonary embolism

**DOI:** 10.1007/s10006-026-01545-5

**Published:** 2026-03-25

**Authors:** Kathi Goldstein, Frederik Holdorf, Martin Lier, Philipp Kauffmann, Phillipp Brockmeyer, Tatjana Khromov, Susanne Wolfer, Norman Moser, Georg Hoene, Henning Schliephake, Boris Schminke

**Affiliations:** 1 Department of Oral and Maxillofacial Surgery, University Medical Center, Goettingen, Germany; 2 Department of Anesthesiology, Emergency and Intensive Care Medicine, University Medical Center, Goettingen, Germany; 3 Department of Clinical Chemistry, University Medical Center, Goettingen, Germany

**Keywords:** Pulmonary embolism, Free flap surgery, Anticoagulation, Head and neck tumors

## Abstract

**Purpose:**

This study investigated the impact of different anticoagulation protocols on the incidence of pulmonary embolism (PE) in patients with head and neck tumors who underwent microvascular flap reconstruction.

**Methods:**

We conducted a retrospective case‒control analysis of 92 patients. Two anticoagulation strategies were evaluated: one group received 10000 IU of unfractionated heparin (UFH) administered over 24 hours (h) starting 6 h postoperatively for one week.The other group received 3 IU of UFH per kilogram of total body weight per h at the onset of surgery until 6 h after surgery. The activated partial thromboplastin time (aPTT) target was subsequently set at 50 to 60 seconds for one week.

**Results:**

The group that received intraoperative UFH presented a significantly lower incidence of PE (6.5% compared with 17.4% in the group that received 10,000 IU of UFH 6 h postoperatively), independent of the type of flap used for reconstruction. This effect was particularly notable in patients who received a forearm flap for reconstruction. There was no statistically significant difference in the incidence of bleeding requiring intervention between the two groups; however, a trend was noted, as postoperative bleeding occurred more frequently in the group receiving intraoperative heparinization.

**Conclusions:**

Our findings suggest that the intraoperative administration of UFH is effective in significantly reducing the risk of PE in patients with head and neck tumors undergoing free flap reconstruction. These results highlight the need for further research to optimize anticoagulation protocols that effectively balance thromboembolic prevention and the risk of bleeding complications.

## Introduction

Head and neck cancer, especially oral squamous cell carcinoma (OSCC), which accounts for approximately 90% of oral malignancies, is a growing global health concern [1–3]. The incidence of OSCC is expected to rise by 40% by 2040, along with increased mortality rates due to prolonged exposure to risk factors [4]. OSCC can lead to significant disfigurement and functional issues, affecting patients’ quality of life [5]. The primary treatment for OSCC is surgery, aiming for complete tumor removal and often requiring careful neck dissection for subsequent microvascular reconstruction of the resulting defect [6, 7]. In rarer instances, microvascular reconstructions are also indicated for ameloblastoma, skin cancer, lip cancer, adenocarcinoma, fibrosarcoma, osteomyelitis, radionecrosis, or drug-related osteonecrosis of the jaw [8–13].

Microvascular free tissue transfer requires an extended operation time for patients [14]. This lengthy procedure carries several risks, including prolonged surgical duration, blood loss, hemorrhagic complications, and potential adverse effects on the overall survival of the free microvascular transplant, as well as thrombotic events. Among these risks, PE is one of the most critical complications associated with free flap surgery [15, 16], especially for patients suffering from cancer [17–19].

PE is an urgent and life-threatening clinical problem caused by emboli that become lodged in the pulmonary arteries and lead to poor oxygen exchange within vital organs. The mortality rate is as high as 30%, while when treated in a timely manner, the rate decreases to 8% [20]. In addition to the abovementioned cancer and immobility due to surgery, the most important risk factors for PE include deep vein thrombosis, a family history of PE, an age over 60 years, smoking, and the use of oral contraceptives or hormone therapy [20, 21]. Therapeutic heparinization is usually initiated. In the event of a fulminant PE with shock, lysis therapy, catheter-based thrombectomy or emergency surgery can be performed [20, 22]. Nevertheless, systemic lysis poses a considerable risk of bleeding complications after major procedures, such as flap surgery [23]. However, the need for anticoagulation in free flap reconstructions is not controversial. Instead, there is controversy as to whether this therapy sufficiently prevents thromboembolism and flap loss or unnecessarily increases the risk of local bleeding and hematoma formation [24]. There are various anticoagulation approaches available, such as the preoperative use of low-molecular-weight heparin or mechanical thromboprophylaxis; unfortunately, the results of using these approaches have not led to the establishment of guidelines [25–28]. In this study, we present our approach for the prevention of PE through the continuous administration of UFH initiated at the beginning of the surgical procedure.

## Methods

### Study population and anticoagulation protocols

The present retrospective case‒control analysis included 92 patients suffering from head and neck tumors who received at least one free flap reconstruction at the Department of Oral and Maxillofacial Surgery at the University Medical Center of Göttingen. Among them, 46 patients did not receive any preoperative or perioperative anticoagulation. However, 6 h post-surgery, 10,000 IU of UFH was administered continuously over a 24-h period for one week via intravenous infusion using a perfusion pump. We subsequently implemented changes in anticoagulation management. The remaining 46 patients received 3 IU of UFH per kilogram of total body weight within one h of the start of the operation, which continued until 6 h postsurgery. The remaining 46 patients received 3 IU of UFH per kilogram of total body weight per hour as a continuous intravenous infusion via a perfusion pump, starting within one h of surgery and continued until 6 h post-surgery. Six hours after the operation, the targeted range of the aPTT was between 50 and 60 s for one week. This protocol is adapted for thrombosis prophylaxis in intensive care patients [29]. The aPTT was monitored four times daily while patients were treated in the intensive care unit and once daily after transfer to the normal ward, with additional measurements performed as required. Patients from both groups received 40 mg of low-molecular-weight heparin daily one week postoperation until they were fully mobilized. The surgery time varied enormously depending on the type of free microvascular transplant used. There were no significant differences in operation time between the two investigated groups. The operation time was determined at the beginning of narcosis and stopped at the end of suturing. All patients with typical symptoms of suspected PE matching the Geneva score were ultimately diagnosed using computed tomography angiography, according to the guidelines. After confirmation of PE, patients were treated according to institutional standards and current guideline recommendations, irrespective of study group allocation [22]. Routine screening for deep vein thrombosis was not performed; diagnostic evaluation and treatment were limited to patients with clinically suspected venous thrombosis. Further details of the patients included in this case‒control study are summarized in Table 1.

**Table 1:** Patient characteristics and operative data

*n* = 92	6 h postop: 10,000 IU UFH/24 h				3 IU UFH and 6 h postop aPTT
Sex			23 female, 23 male	23 female, 23 male	
Mean age (min; max)		66 years (15; 89)			66,1 years (37; 91)
Mean surgery time (min; max)		9,9 h (6,1; 18,1)			9,5 h (4,2; 15,1)
Forearm flap		16 Patients			16 Patients
Fibula flap		14 Patients			14 Patients
Anterolateral thigh flap		12 Patients			12 Patients
Scapula flap		4 Patients			4 Patients

**Fig. 1:**
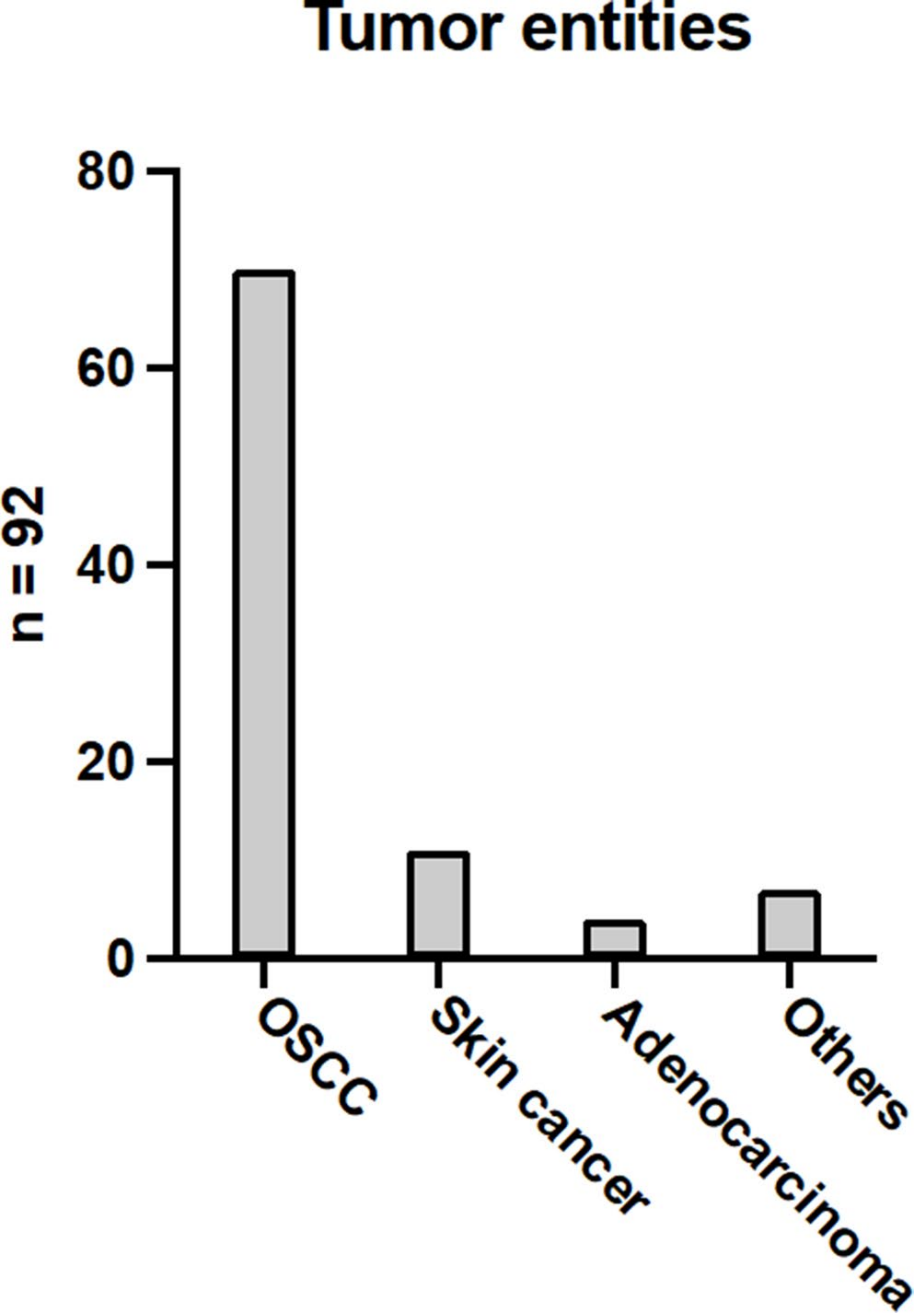
Tumor entities. A total of 92 patients were diagnosed with tumors in the head and neck region. Among them, 70 patients (76.1%) suffered from OSCC. Eleven patients (12.0%) had a type of skin cancer. Four patients (4.3%) were diagnosed with adenocarcinoma. Additionally, seven patients (7.6%) suffered from various rare tumors

A total of 92 free flap reconstructions were documented. The data were collected based on a prospectively managed, computer-supported register in which the basic data of all patients, details of the procedure carried out, and information on success and possible complications were recorded. The distribution of tumor entities is shown in Fig. 1. The majority of the patients, accounting for 76.1%, exhibited OSCC. Among those who received free microvascular transplants, skin tumors were present in 12% of patients, whereas those with adenocarcinomas constituted 4.3%. Additionally, tumors classified as “other,” including odontogenic tumors, fibrosarcoma, and lip carcinoma, were less common, accounting for 7.6% of the cases.

**Fig. 2:**
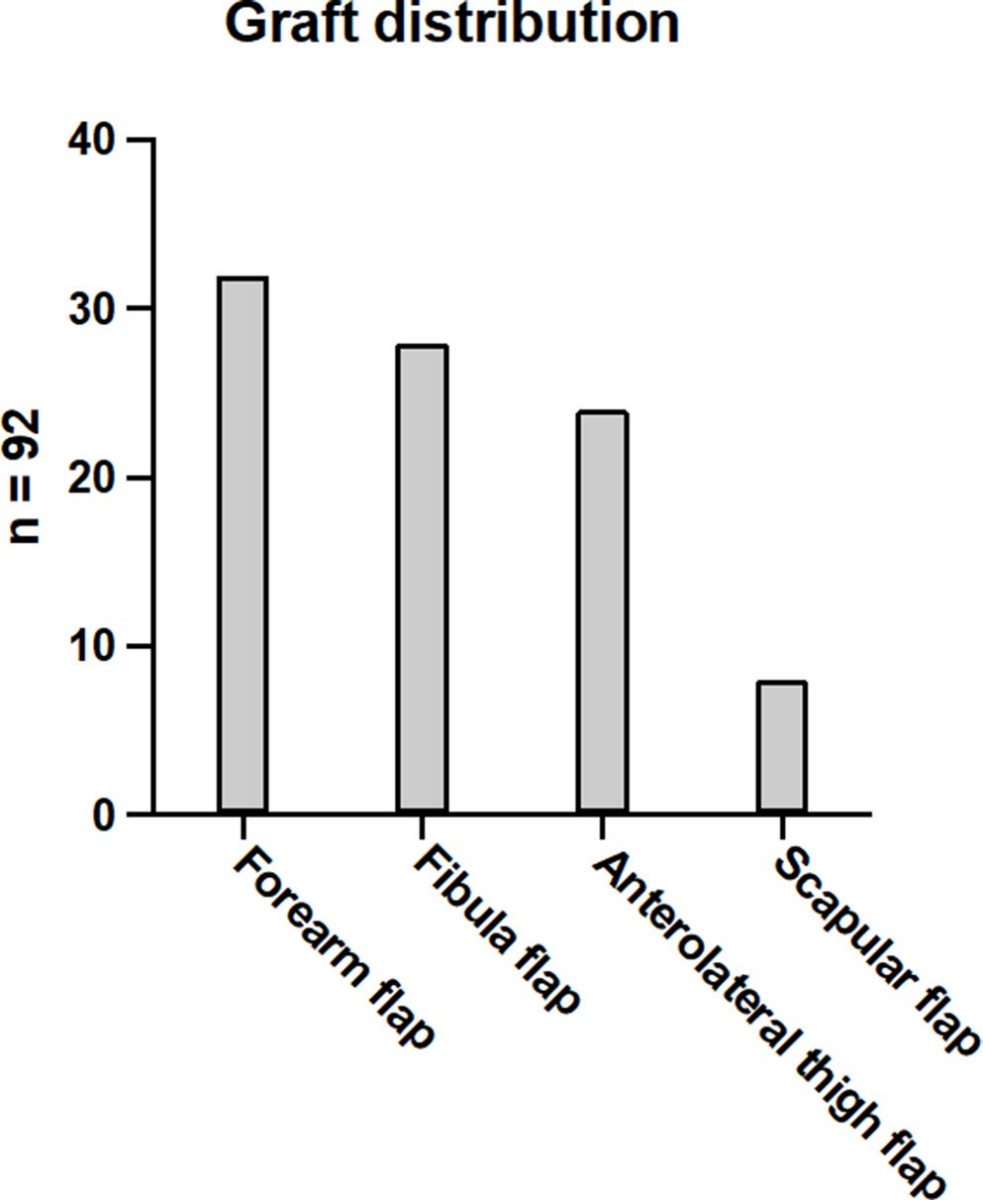
Graft distribution: A total of 92 free microvascular transplants were performed in our clinic during the study period. From left to right, 32 patients (34.8%) received forearm flap surgery. Twenty-eight patients (30.4%) received fibula flap surgery. Twenty-four patients (26.1%) received anterolateral thigh flap surgery. Finally, eight patients (8.7%) received a scapula graft%

The distribution of free microvascular grafts for defect reconstruction is shown in detail in Fig. 2. In each comparison group, the same number of patients was included, depending on the type of microvascular graft used and the two anticoagulation protocols, as is required in a case‒control study. A radial forearm flap was used most frequently in 34.8% of the cases for the reconstruction of defects of soft tissue only. The free fibula flap was used most often in 30.4% of cases for defect reconstruction of soft and hard tissue in the head and neck region. For the reconstruction of larger soft tissue defects, for example, at the tongue or after extraorally perforated OSCC, an anterolateral thigh flap was used in 26.1% of cases. The scapular flap was used for extensive composite defects with large soft tissue requirements or for reconstruction of the midface area in 8,7% of cases.

### Statistical analysis

We considered the effect size, significance level and statistical power when determining the sample size and concluded that the selected sample of 92 patients was adequate to detect a significant difference. Patient data were collected using Microsoft Excel (2016) and analyzed with GraphPad Software, Inc. (2024).

The comprehensive analysis of all flap surgeries revealed a significant difference in survival curves between the two groups, as demonstrated by the log-rank test (Chi² = 4.100; *p* = 0.0429). This finding indicates a differential cumulative incidence of PE over the observation period. The hazard ratio (HR) was 3.506 (95% confidence interval: 1.127–10.91), indicating that the risk of PE in the group receiving only 10,000 IU of UFH continuously over 24 h starting at 6 h postsurgery was more than three times greater.

In a focused subgroup analysis of patients receiving radial forearm flap surgery, a considerably greater difference was noted between the groups. Both the log-rank test (*p* = 0.0162) and the Gehan-Breslow-Wilcoxon test (*p* = 0.0193) achieved statistical significance, suggesting a clear distinction in event-free survival between the groups. The significance of both tests indicates that the observed differences not only persisted over time but also manifested early in the postoperative period. Moreover, the Mantel‒Haenszel model yielded an HR of 5.776 (95% CI: 1.383–24.12), and the log-rank model yielded an HR of 8.123 (95% CI: 2.024–32.60). Consequently, in the group receiving only 10,000 IU of UFH continuously over 24 h, starting at 6 h postsurgery, the risk of PE increased by up to eightfold, underscoring the clinical relevance of the findings.

Additionally, Kaplan‒Meier analyses were performed to determine embolism- and bleeding-free postoperative survival. For all the statistical tests, the significance level was set at α < 0.05.

## Results

**Fig. 3:**
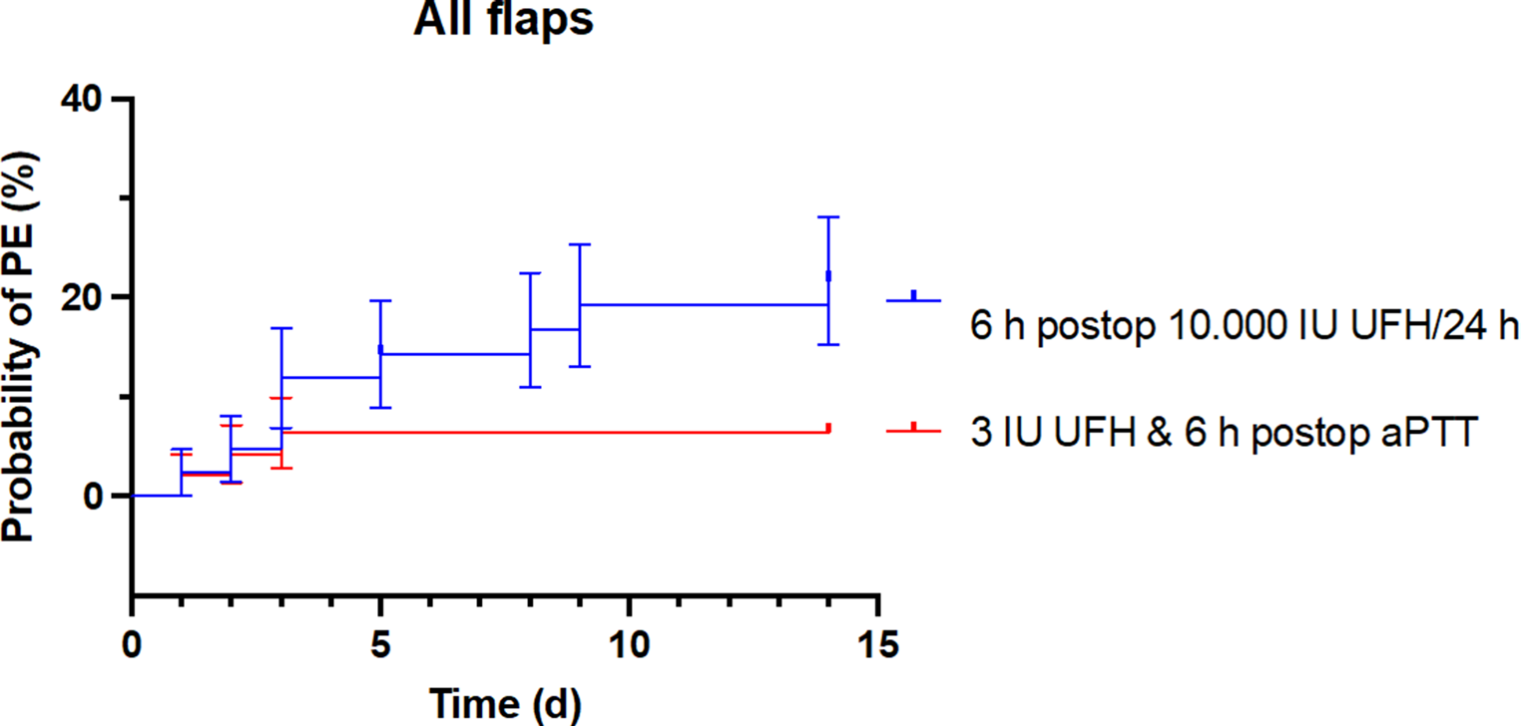
Probability of PE (%) on the Y-axis as a function of time in days (d) after surgery on the X-axis. The red line represents the new treatment regimen that incorporates intraoperative UFH administration, whereas the blue line depicts the patient cohort receiving 10,000 IU of UFH postoperatively over a 24-h period, starting 6 h after surgery. The analysis revealed a significantly lower probability of PE, regardless of the type of flap used, for patients who received the new regimen with intraoperative UFH administration (*p* = 0.04)

This first section presents a comprehensive overview of the 92 patients included in the study, with 46 patients in each treatment group, as illustrated in Fig. 3. The analysis revealed a significantly lower probability of PE (*p* = 0.04) among patients who received 3 IU of UFH per kilogram of total body weight per h intraoperatively, with the aPTT maintained at 50 to 60 s until 6 h postoperatively (indicated by the red line), than in the group that received 10,000 IU of UFH 6 h postoperatively (indicated by the blue line).

**Fig. 4:**
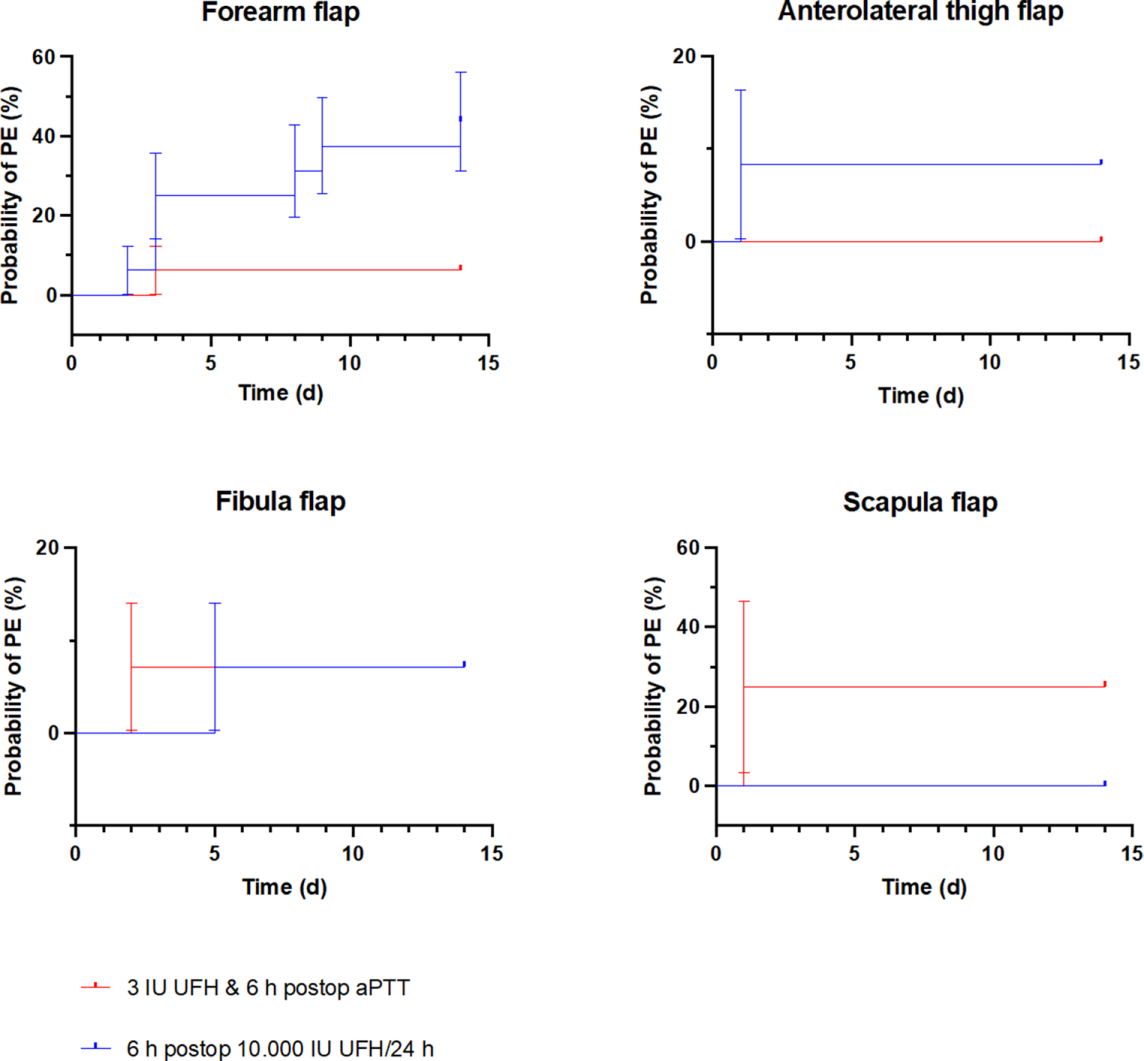
Probability of PE (%) based on the type of flap utilized, plotted on the Y-axis, as a function of time in days postsurgery on the X-axis. The red line represents the new treatment regimen that includes intraoperative administration of UFH, whereas the blue line depicts the patient cohort receiving 10,000 IU of UFH postoperatively over 24 h, starting 6 h after surgery. Top left: Analysis of the forearm flap indicates a significantly lower probability of PE in patients who received the new protocol with intraoperative UFH (*p* = 0.03). Bottom left: No significant differences were noted for the fibula flap surgery. Top right: No significant differences were observed in the group that received an anterolateral thigh flap surgery. Bottom right: No significant differences were found in the group that received a scapula flap surgery

To provide a more detailed analysis of the occurrence of PE, the patients were categorized according to the type of microvascular flap used, as shown in Fig. 4. Sixteen patients received forearm flap surgery under the previous protocol, while another 16 patients received forearm flap procedures according to the new anticoagulation regimen. The analysis revealed a significantly lower incidence of PE among patients treated with the new protocol, which included intraoperative administration of UFH (Fig. 4; top left). In contrast, no differences were observed in patients receiving free fibula flap surgery, where 14 patients each from the old and new protocols showed comparable outcomes (Fig. 4; bottom left). Evaluations of the anterolateral thigh flap surgery, with 12 patients in each group (Fig. 4; top right), and the scapula flap surgery, with 4 patients in each group (Fig. 4; bottom right), revealed trends without statistical significance.

**Fig. 5:**
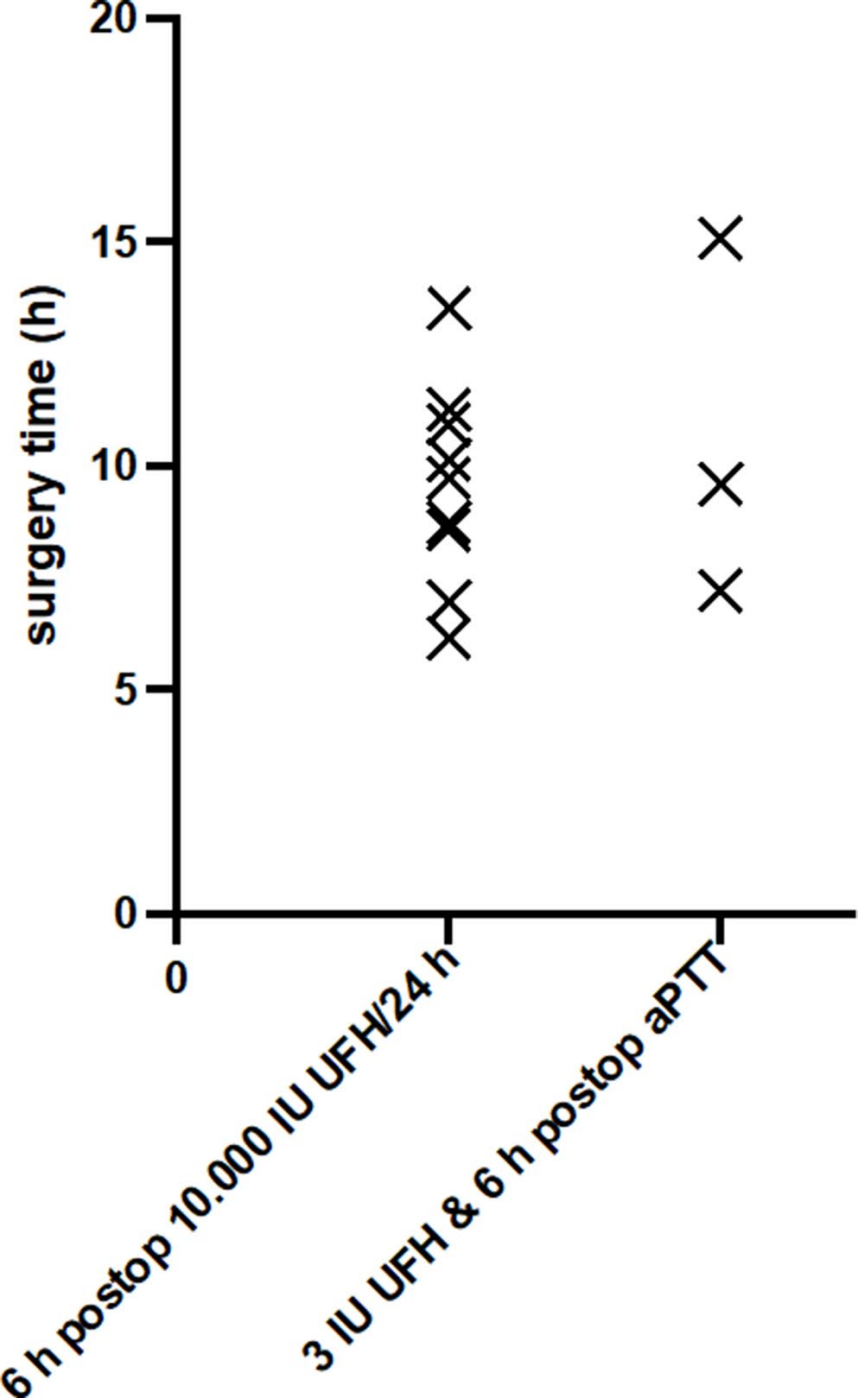
Distribution of surgery time. Surgical time (h) on the Y-axis as a function of all patients (dots) on the X-axis. Patients with PE are marked with a cross on the x-axis. The red dots represent the new treatment regimen that incorporates intraoperative UFH administration, while the blue dots depict the patient cohort receiving 10,000 IU of UFH postoperatively over a 24-h period, starting 6 h after surgery

Interestingly, there was no statistically significant correlation between a long operation time and the incidence of PE in either group studied (Fig. 5). The graph clearly demonstrates that PE can occur at any operation time, regardless of whether it is long or short.

**Fig. 6:**
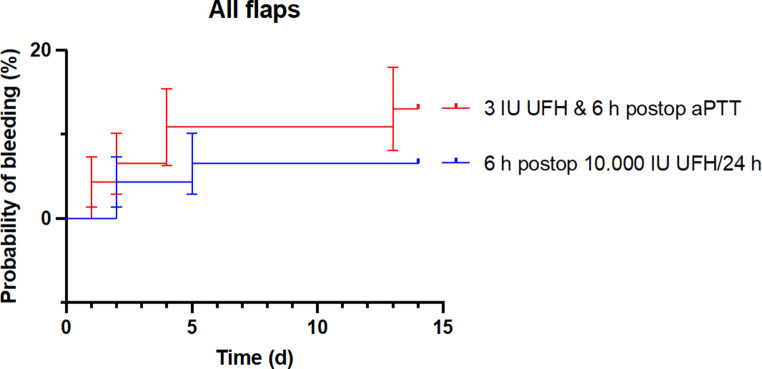
Probability of bleeding (%) on the Y-axis as a function of time in days (d) after surgery on the X-axis. The red line shows the new treatment regimen with intraoperative UFH administration. The blue line shows the patients who received 10,000 IU UFH over 24 h, starting 6 h postoperatively. The analysis revealed no significant differences in the probability of bleeding between the groups (*p* = 0.6)

Figure 6 illustrates the probability of bleeding, a logical consequence of anticoagulation treatment, for the two patient cohorts investigated. The comparison revealed no significant difference in the incidence of bleeding that required intervention during the inpatient observation period. However, a trend toward increased bleeding was observed in the group that received intraoperative UFH.

The procedure-related complications are summarized in Table 2, highlighting the differences between the two anticoagulant treatment approaches. In the cohort of 46 patients who received intraoperative administration of 3 IU of UFH per kilogram of total body weight per h, with a target aPTT of 50 to 60 s 6 h after surgery, the complication rate of PE was significantly lower (6.5%) than that of the other groups. However, the incidence of postoperative bleeding requiring surgical intervention increased from 4.3% to 13%, although this change did not reach statistical significance.

**Table 2:** Procedure-related complications

Complications	6 h postop: 10,000 IU UFH/24 h	3 IU UFH and 6 h postop aPTT	
Pulmonary embolism^*^	17.4% (8/46)	6.5% (3/46)	
Secondary bleeding	4.3% (2/46)	13% (6/46)	
Revision anastomosis	15.2% (7/46)	5.4% (2/46)	
Loss of graft	4.3% (2/46)	6.5% (3/46)	

*Significant difference (*p* > 0.05)

Fortunately, the revision rate of vascular anastomoses in the group that received intraoperative UFH decreased to 5.4% compared with that in the other group that received only postoperative UFH, which had a revision rate of 15.2%. Although these results did not reach statistical significance, they showed a clear trend. No significant differences were detected regarding graft loss.

## Discussion

The aim of our retrospective case‒control study at a single institution was to compare two anticoagulation regimens and, on the basis of the results obtained, reduce the incidence of our primary complication, PE, following free microvascular flap surgery [30]. Our patient cohort and the procedures performed are largely consistent with global data and standards [31], making them easily comparable.

Patients who receive major oral and maxillofacial surgeries with free flap reconstruction are at high risk for pulmonary complications, such as PE [32]. Therefore, continued research is essential to mitigate these risk factors [33]. However, the use of anticoagulation agents during extensive surgical procedures is a contentious issue because of concerns over the potential for increased bleeding, particularly during tumor resection [24, 34].

Preoperative anticoagulation with low-molecular-weight heparin in patients with head and neck tumors has been shown not to significantly increase the risk of bleeding, which is consistent with our findings regarding postoperative bleeding [25]. Although an increased risk of hematoma formation associated with continuous unfractionated heparin infusion has been reported in other clinical settings [28], this was not observed as a statistically significant finding in our cohort. In contrast, several extensive reviews and meta-analyses have reported conflicting results, suggesting a potentially increased bleeding risk [35, 36].

Unlike other studies, the data presented here suggest a potential effect of early anticoagulation on the incidence of PE. A study examining outcomes following microvascular free tissue transfer reported a lower incidence of adverse medical events, such as PE, than did the literature; however, an increase in 30-day mortality was also observed [16]. Consequently, the significance of PE is widely recognized, and careful monitoring in an intensive care setting is recommended for these patients [37]. While intraoperative administration of UFH may significantly reduce the incidence of PE, it does not completely eliminate the risk, suggesting that other factors may be more influential in the development of PE [38–40].

The direct association between the occurrence of PE and the use of a forearm flap in this study remains unclear. The current literature on this topic is limited; however, a case report has been published concerning OSCC in which a PE occurred following reconstruction of the oral cavity and oropharynx using a forearm flap [41]. It can only be speculated whether the abduction of the arm during positioning or lifting of the flap might play a role in the development of thrombosis. However, it is clear that the intraoperative administration of UFH reduces the occurrence of the ultimate consequence of venous thrombosis, namely, PE. The lack of a significant effect in the free fibula flap subgroup may be attributable to prolonged lower limb immobilization, delayed postoperative mobilization, and limited statistical power due to the small subgroup size [28, 42, 43].

Although not statistically significant, there was a trend toward a reduction in the number of revision surgeries for anastomoses with the intraoperative administration of UFH. These data are consistent with similar approaches previously considered in hand surgery for vascular abnormalities [44]. Conversely, the intraoperative administration of anticoagulants has been shown to offer no additional benefit for free flap surgery survival during anastomosis revision [34]. Overall, further data on revision procedures for anastomoses should be collected.

In contrast to our findings regarding operation time and the lack of a correlation with PE, a substantial study indicates a generally increased incidence of deep vein thrombosis, which can lead to PE, associated with prolonged surgical duration [45]. Their study investigated the prevention of perioperative venous thromboembolic complications using pneumatic compression devices in patients with OSCC and adopted a fundamentally different approach by omitting anticoagulation. The combination of both strategies may prove to be more advantageous [46].

We consider the new regimen involving intraoperative heparinization to be our new standard and will continue to collect data on an ongoing basis, particularly to assess whether side effects such as heparin-induced thrombocytopenia [47–49] may play a significant role. To date, no cases of heparin-induced thrombocytopenia have been observed.

## Conclusion

Our findings clearly indicate that the incidence of PE is significantly reduced by administering 3 IU of UFH per kilogram of total body weight per h at the onset of surgery, continuing for a total of 6 h postoperatively, followed by maintaining the aPTT at 50 to 60 s for one week. In comparison, the group receiving only 10,000 IU of UFH continuously over 24 h, starting 6 h post-surgery, has a substantially higher risk of PE. Consequently, across all flap surgeries the probability of PE is reduced by threefold. In particular, for forearm flap surgery the risk is diminished by as much as eightfold. Importantly, there was no significant increase in the risk of bleeding associated with the intraoperative administration of UFH; however, a trend towards increased postoperative bleeding was observed. Therefore, further research, along with careful monitoring of bleeding complications, is essential to optimize anticoagulation protocols that achieve an appropriate balance between thromboembolic prevention and bleeding risk.

## Data Availability

The original data are available from the corresponding author upon reasonable request.
